# Impact of age, antiretroviral therapy, and cancer on epigenetic aging in people living with HIV

**DOI:** 10.1002/cam4.5809

**Published:** 2023-03-23

**Authors:** Yulan Qing, Ricky Chan, Pingfu Fu, Jennifer Cullen, Alexander Miron, Jeffrey M. Jacobson, John Pink, Stanton L. Gerson

**Affiliations:** ^1^ Case Comprehensive Cancer Center Case Western Reserve University Cleveland Ohio USA; ^2^ Department of Population and Quantitative Health Sciences Case Western Reserve University Cleveland Ohio USA; ^3^ Department of Genetics and Genomics Case Western Reserve University Cleveland Ohio USA; ^4^ Division of Infectious Diseases and HIV Medicine Case Western Reserve University Cleveland Ohio USA

**Keywords:** antiretroviral therapy, cancer, epigenetic aging, HIV

## Abstract

**Background:**

Premature aging has been identified as a global risk factor for cancer. Causes of premature aging are multifactorial, including inflammation, infection, chronic stress, and lifestyle factors.

**Method:**

We evaluated whether premature aging in people living with HIV (PLWH) was associated with antiretroviral therapy (ART) or the diagnosis of cancer. We used well‐established DNA methylation patterns to assess premature aging, using Horvath et al., in individuals with HIV located in Cleveland, Ohio and compared these to standardized datasets of US historical blood samples. Some of the PLWH developed cancer over time.

**Results:**

We found that DNA methylation analysis identified accelerated aging in PLWH whereas ART therapy mitigated the advancement of DNA methylation age. A variety of cancers were observed in this population, but a cancer diagnosis was not significantly associated with more advanced DNA methylation age.

**Conclusion:**

We find that the age acceleration detected in PLWH is mitigated by ART therapy and is not further accelerated by a diagnosis of cancer.

## Graphical abstract

People living with HIV experience age acceleration based on DNA methylation analysis that isameliorated by antiretroviral treatment (ART), either early (ART1) or later (ART2) in the development ofantiretroviral treatment regimens. However, while PLWH experience an increase in the number ofmalignancies, those malignancies are not associated with an additional burden of age acceleration nor do theyrepresent a subgroup with excessive age acceleration.

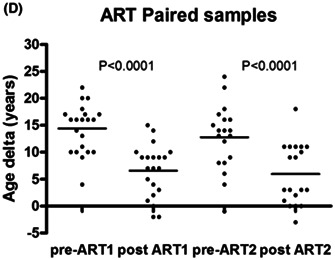

## 1 INTRODUCTION

The introduction of highly active antiretroviral therapy (HAART or ART) in the mid‐1990s, has reduced the number of deaths from AIDS, and increased overall survival. People living with HIV (PLWH) are living much longer and the number of older individuals living with HIV is increasing. In 2019, an estimated 45% of Americans living with HIV were aged 55 and older, 27% were aged 60 and older, and 6% were aged 70 and older.^1^ Though ART lowered the risks of developing AIDS‐defining cancers, including Kaposi's sarcoma and non‐Hodgkin lymphoma (NHL), the incidence of non–AIDS‐defining cancers (NADCs) rose by more than threefold.^2^, ^3^ Clinical epidemiologic research also suggests a higher prevalence of other age‐associated comorbidities in PLWH. These include earlier onset cardiovascular disease, bone fractures, osteoporosis, kidney and liver disease, cognitive decline, and frailty.^4^, ^5^, ^6^

ART therapy targets several aspects of HIV replication. As these therapies have evolved, ART treatment can be differentiated into two eras, era I (1996–2001) and era II (after 2002).^7^, ^8^, ^9^, ^10^ The current classes of drugs in antiretroviral therapies include: nucleoside reverse transcriptase inhibitors (NRTIs), non‐nucleoside reverse transcription inhibitors (NNRTIs), protease inhibitors (PIs), and integrase inhibitors (INSTIs). The most commonly used regimens currently include two NRTIs plus an INSTI. While these medicines inhibit HIV replication, they also cause cell, organ, and systemic side effects.^11^, ^12^ ART greatly increased the survival of PLWH, however, long‐term complications of HIV infection, including premature aging, are multifactorial and might be related to the virus itself or to adverse effects of ART.^5^, ^13^

As objective measures of premature aging, changes in DNA methylation have been shown to be the most reliable biomarker of age.^14^, ^15^ Several epigenetic clocks have been generated based on DNA methylation patterns that are reliable estimators of biological age. The first generation clocks such as those described by Horvath and Hannum, were developed to assess the biological age of multiple tissues^16^ or blood samples.^17^ The second generation clocks, such as PhenoAge and GrimAge, have incorporated clinical parameters with DNA methylation profiles to predict aging‐related phenotypes, including mortality.^18^, ^19^

PLWH have been observed to display premature or accelerated aging at the molecular level. Using first generation DNA methylation clocks, analysis from several groups revealed a 5‐year increase in biological age in PLWH as compared to uninfected individuals.^20^, ^21^, ^22^ While ART provides effective inhibition of HIV replication in most subjects, the impact of these treatments on epigenetic age acceleration is unclear. Recently, studies have found that ART leads to a partial reduction in epigenetic age acceleration after 2 years of continuous therapy.^23^

Age is a major risk factor for cancer development and the majority of cancer patients are 65 years or older. Cancer and its associated therapies can also influence the aging process. Cancer survivors are prone to age‐related diseases and comorbidities earlier than members of the general population.^24^, ^25^, ^26^, ^27^ The correlation of epigenetic aging and cancer has also been studied and epigenetic clocks have been used to assess cancer risk.^28^, ^29^ Epigenetic age in peripheral blood samples is statistically higher in survivors of childhood cancer than in noncancer controls.^30^, ^31^ Significant increases in epigenetic age acceleration were identified posttreatment in breast cancer patients undergoing surgery followed by radiotherapy or chemotherapy compared to pretreatment.^32^

To determine if PLWH with epigenetic age acceleration have an increased incidence of cancer, we asked whether age acceleration resulting from HIV infection was slowed by ART, and if so whether the slowing improved with extended treatment duration. We also investigated whether there was evidence that PLWH who develop cancer have greater evidence of age acceleration or less benefit from ART in the mitigation of age acceleration.

## 2 METHODS AND EXPERIMENTAL DESIGN

### 2.1 Study subject and biospecimen identification

Cryopreserved peripheral blood samples were obtained from the Case Western Reserve University Center for AIDS Research (Case CFAR) biorepository.^33^ Study subjects (n = 96) known to be PLWH and aged >30 years old were identified and their samples acquired following approval by the IRB of the University Hospitals of Cleveland. Patients followed under clinical observation were entered into the database between the years 1989 and 2021. Demographic and clinical data were acquired from subjects for whom DNA could be obtained. This included patient's chronologic age (years), race (Black/White), sex (male/female), viral loads, CD4 counts, ART treatment types and dates, cancer diagnosis date and type, and smoking history (nonsmoker, smoker, and past smoker). The first recognized HIV infection date and smoking history are self‐reported by patients while other information was obtained from medical records.

### 2.2 DNA preparation

DNA was purified from whole‐blood samples using the Maxwell 16 LEV Blood DNA Kit (Promega Inc., Madison, WI) and quantitated using the Broad Range Qubit method (Invitrogen Inc., Waltham, MA).

### 2.3 Methylation array

The Illumina MethylationEPIC BeadChip is a comprehensive array that covers over 850,000 methylation sites quantitatively across the genome at single‐nucleotide resolution. It is a PCR‐free protocol that requires 250–1000 ng DNA input using the Infinium HD Assay. Genomic DNA is quantified by Qubit Fluorometer (Invitrogen) and qualified by agarose gel. Only DNA of high quality was selected and normalized to 25 ng/uL. Following a three step reaction, samples are bisulfite converted using the Zymo Research EZ DNA methylation kit. Once bisulfite conversion was complete, the Infinium HD protocol is followed. First, the DNA is amplified, fragmented, and precipitated to prepare for hybridization to the Beadchip. Twelve samples can be loaded onto one Beadchip. Single‐base extension of the oligos on the BeadChip is performed using the bisulfite‐converted DNA as a template. Beadchips are then scanned on the Illumina iScan or NextSeq systems. All raw data, along with corresponding manifest file, decode file, and sample sheet are uploaded into Illumina's GenomeStudio Methylation software module. Internal controls are checked within the software to validate complete bisulfite conversion and successful processing of the Infinium HD assay for each sample. Data are then ready for downstream analysis.

### 2.4 Estimation of DNA methylation age and age delta

The DNA methylation (DNAm) age was calculated based on the algorithms developed by Horvath et al,^16^ using whole‐blood DNA samples. The ratio of intensities between methylated and unmethylated alleles (beta values) was determined for each CpG probe site, and the beta values were used to estimate the DNAm age. An “age delta” was calculated as the difference between the DNAm age and the chronologic age of each patient at the time the sample was analyzed.

For a subset of 40 patients from this cohort, a second blood sample was retrospectively acquired from a time at least 10 years prior to their current sample and prior to the initial ART treatment. DNA methylation age was analyzed at both time points to allow for the comparison of DNAm age before and after the initiation of ART.

### 2.5 Study endpoints

Age delta was observed to be normally distributed and therefore modeled as a continuously distributed study outcome.

### 2.6 Statistical analysis

Descriptive statistics were computed for the overall study cohort for both categorical and continuous patient characteristics. Formal comparisons of the age delta were made using the Students t‐test for dichotomous factors, and analysis of variance (ANOVA) for categorical variables with three or more levels. Histograms were constructed to confirm normality in distributions of all age‐related variables (chronologic age, and age delta). Pearson and Spearman rank correlation analysis was performed to assess the association and directionality of all age‐related variables. The effect of each factor on age delta was determined in the univariate analysis. As the effects of demographic factors are of interest for clinical studies and the effect of ART treatment is of specific interest of HIV/AIDS studies, we included those variables in the multivariable regression model even though they were not significant (except for age) in the univariate analysis. The effects of age, race, gender, and ART treatment on age delta were further evaluated using a multivariable linear regression model. All tests were two‐sided and p <0.05 were considered statistically significant.

## 3 RESULTS

### 3.1 Patient population

Demographics of patients are noted in Table 1. For the full cohort of 96 patients, the median age at blood collection was 50.5 years, and the average time from diagnosis to ART treatment onset was 1.3 years. In this group, 55 started with the era I ART therapy and 41 started treatment with era II ART therapy. At the time of blood collection, the viral loads of most patients (93/96) were approximately 40 copies/mL, while the viral loads of 3/96 were over 100 copies/mL. CD4 counts of 3/96 were below 200 cells/mL, while 69/96 were above 500 cells/mL. There were 35 cancers observed with skin lesions being most common, followed by anal cancer.

**TABLE 1:** Descriptive characteristics of the study cohort (*N* = 96).

	Median (±SD), range
Continuous variables	
Chronologic age, years	50.5 (±10.5), 28–69
Time from HIV to ART, years	1.3 (±4.5), 0–19
Viral load, (copy/mL)	60 (±303), 20–2973
CD4, (count/mL)	743 (±371), 109–2150
Categorical variables	*N* (%)
Sex	
Male	88 (91.7%)
Female	8 (8.3%)
Race	
White	80 (83.3%)
Black	16 (16.7%)
Cancer diagnosis	35 (36.5%)
Type of cancer (*n* = 35)	
Skin	11 (11.46%)
Anal	7 (7.29%)
Leukemias/Lymphomas	4 (4.2%)
GU	4 (4.2%)
Lung	3 (3.1%)
Kaposi's sarcoma	3 (3.1%)
Colorectal	2 (2.1%)
Esophageal	1 (1.0%)
Smoker	
Nonsmoker	31 (32.3%)
Smoker	21(21.9%)
Past smoker	43(44.8%)
Unknown	1 (1.0%)
Initial ART era	
First (I)	55 (57.3%)
Second (II)	41 (42.7%)

### 3.2 Association of HIV infection and DNA methylation age advancement

**FIGURE 1:**
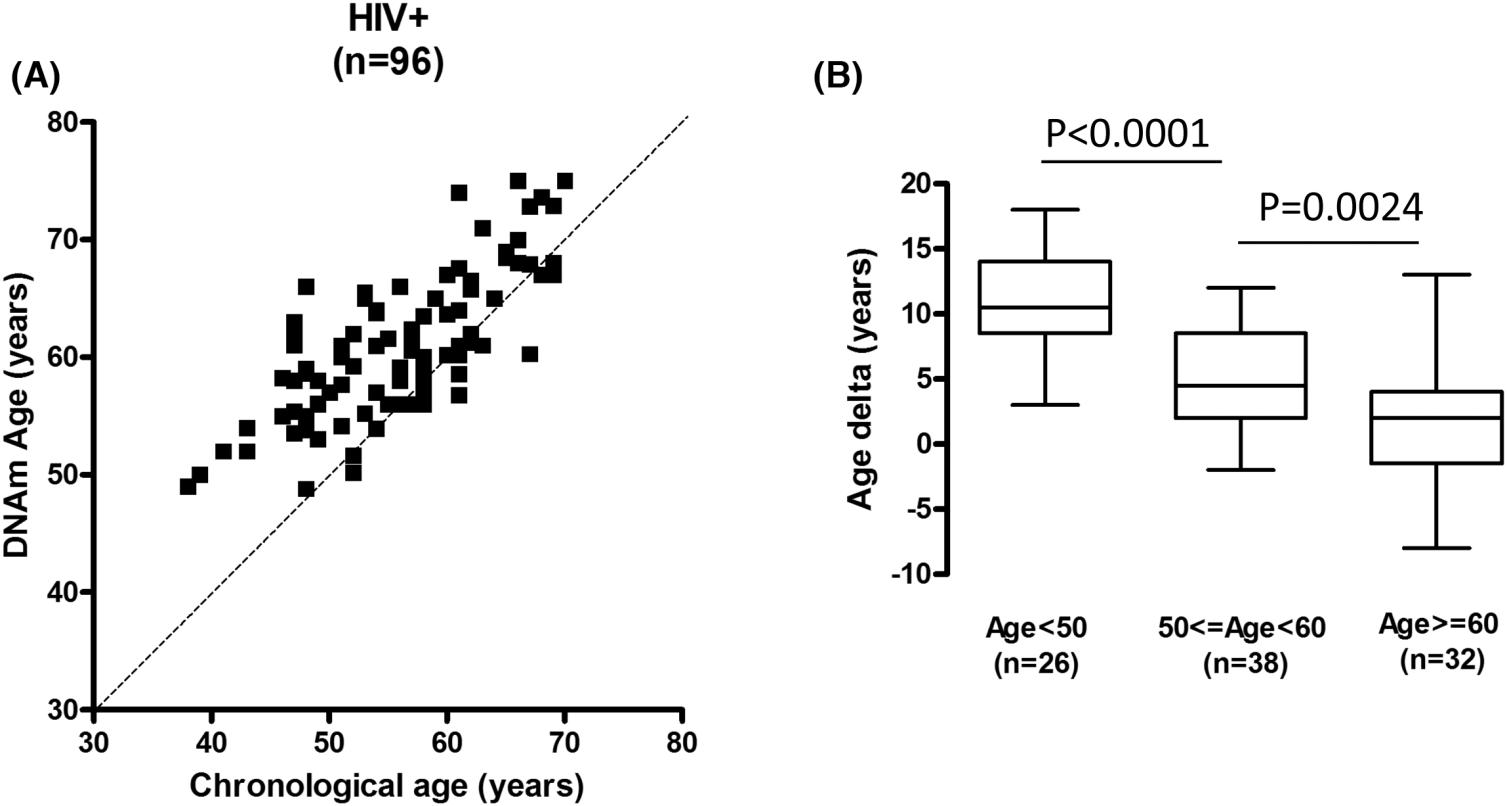
(A) Scatter plot of DNAm age versus chronologic age of PLWH (*n* = 96). Dashed line indicates diagonal, DNAm age = Chronologic age. (B) Age delta by chronologic age category. Three age groups in (A) are shown: individuals younger than 50 years old, individuals between 50 and 59 years old, and individuals at least 60 years old.

To examine whether epigenetic age was advanced in PLWH, genome‐wide methylation profiling of PBMC DNA from each individual was performed, DNAm age was calculated, and the DNAm age versus chronologic age of each sample was plotted. The DNAm age of a majority of the samples (78/96) was older than the chronologic age of the patient (Figure 1); the mean age delta (or accelerated age) was 5.3 ± 5.1 years. Samples from younger individuals displayed more advancement of DNAm age (greater age delta) than samples from older individuals (Figure 1B).

### 3.3 DNAm age advancement in the ART eras

**FIGURE 2:**
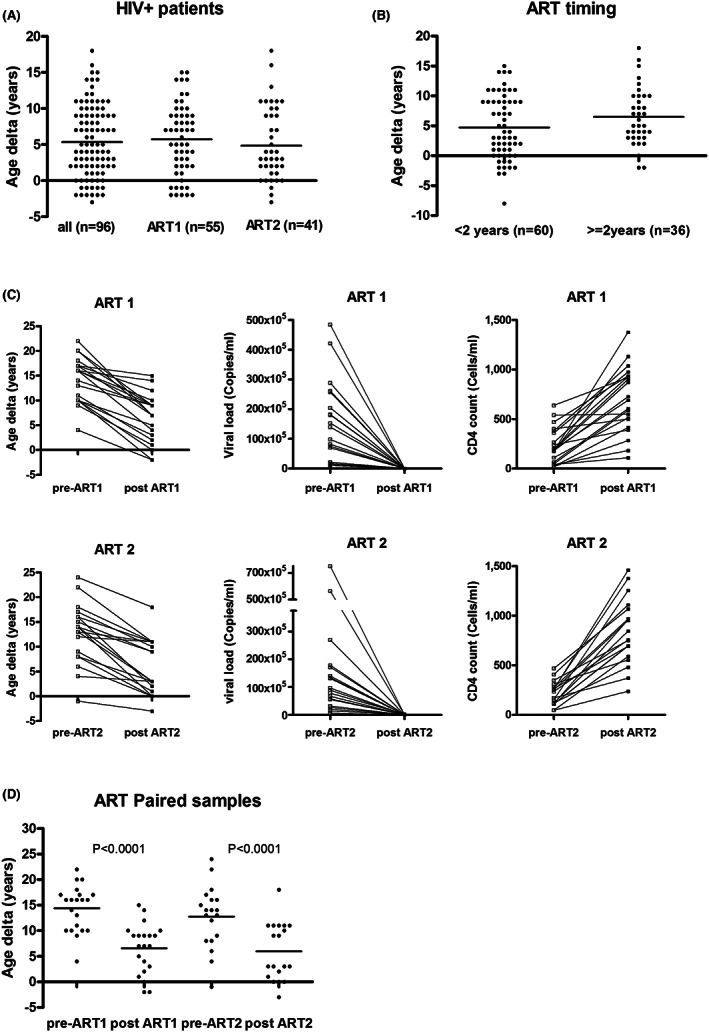
(A) Effects of ART era on age delta. PLWH are divided into two groups based on the initial ART treatment (ART1 or ART2); age deltas are shown for each group. (B) Effects of ART initiation timing on age delta. PLWH are divided into two groups based on the interval of HIV diagnosis and ART initiation; age deltas are shown for each group. (C) Effects of ART on age delta of PLWH. PB samples were retrospectively obtained from a subset of PLWH started with either ART1 (*n* = 21) or ART2 (*n* = 19) treatments. Pre‐ART indicated the time of diagnosis, without ART treatment, post‐ART indicated the time at least 10 years after ART initiation. The lines represent age deltas before–after ART treatment. Viral loads (HIV RNA copies/mL) and CD4 counts (cells/mL) at the time of sample collection were shown in the graph. (D) Age deltas of before–after of each group are shown in (C).

In the CFAR care guidelines, patients diagnosed with HIV are treated with the best ART therapy available. As HIV treatment evolved, patients who started with era I ART later transitioned to era II ART. All the patients in this study are currently being treated with era II ART. We examined whether age delta among PLWH was affected by their initial ART therapy. In this cohort of patients, we found that samples from patients initially treated with era I ART displayed similar age deltas to those initially treated with era II ART (5.8 ± 4.8 years vs. 4.8 ± 5.5 years, *p* = 0.34) (Figure 2A).

Next, to address whether delays in initiation of ART treatment affected DNAm age of PLWH, PLWH were divided into two groups based on the interval from diagnosis to initiation of treatment. For this analysis, patients were divided into immediate treatment (<2 years) or delayed treatment (≥2 years). The age delta was not significantly different between these groups, 4.7 ± 0.7 years for the immediate treatment group, (*n* = 60), and 6.5 ± 0.8 years for the delayed treatment group, (*n* = 36, *p* = 0.10) (Figure 2B).

To assess whether ART contributed to premature aging in PLWH, the age deltas of PLWH were compared before and after ART therapy. We retrospectively identified 40 individuals from this cohort, whose blood samples prior to ART treatment were available. Paired samples were obtained, one at the time HIV infection was confirmed (prior to ART treatment), and the other at least 10 years after initiation of ART treatment. Among these 40 patients, 21 started with era I ART and 19 started with era II ART. All the individuals showed decreased viral loads and increased CD4 counts after treatment. In these individuals, age acceleration markedly lessened from a mean of 13.6 ± 5.3 years at diagnosis to 6.3 ± 5.2 years after more than 10 years of therapy, and in no instance did age delta increase (Figure 2C,D). This observation indicates that both ART treatments reverse the advancement of DNA methylation age.

### 3.4 DNAm age and cancer patients with HIV infection

**FIGURE 3:**
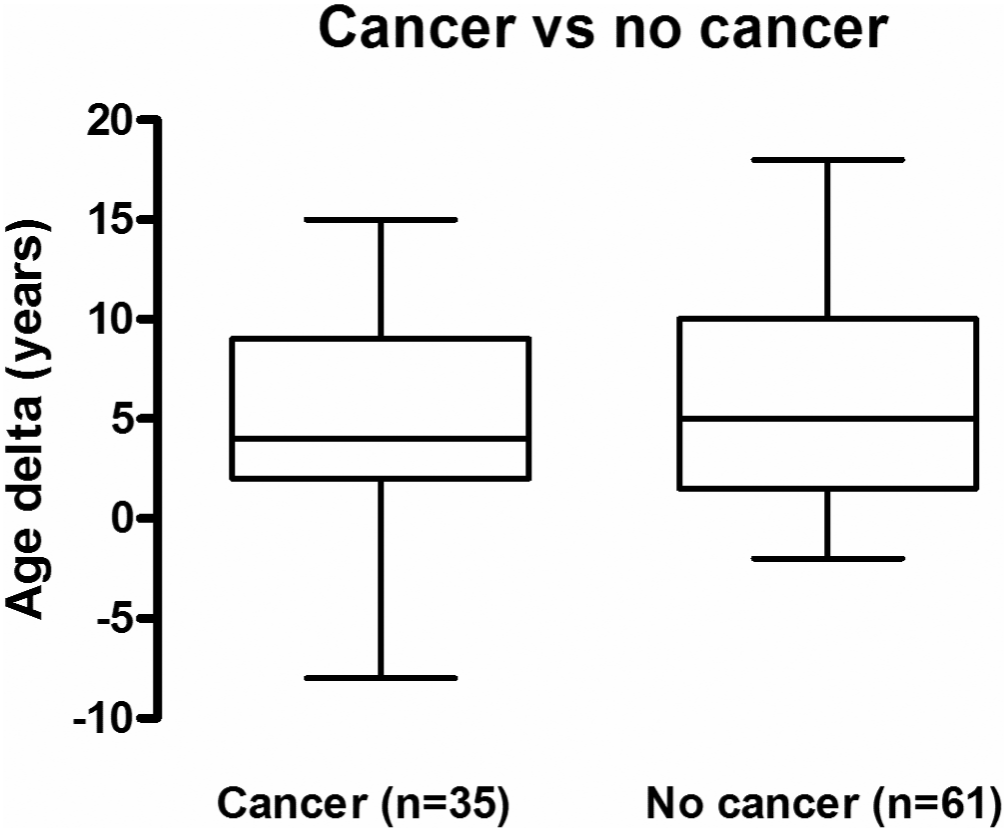
Age delta and cancer patients with PLWH. PLWH are divided into two groups based on cancer occurrence; age deltas are shown for each group.

It is currently unknown whether HIV infection causes an advancement of DNAm age that subsequently leads to cancer development or whether the development of cancer will accelerate the advancement of DNAm age among PLWH. In this cohort of 96 PLWH, 35 developed cancer (Table 1). Most cancers (27/35) were diagnosed after HIV diagnosis, a few cancers (5/35) were observed at the time of HIV diagnosis, and two patients were diagnosed with cancer prior to their HIV diagnosis (Table S1). Collectively, the mean age delta of cancer patients was similar to that of patients without cancer—(4.6 ± 0.9 years, *n* = 35 vs. 5.8+/−0.7 years, *n* = 61, *p* = 0.30) (Figure 3). Among cancer types, some appeared to show greater evidence of age acceleration (e.g., lung cancer and Kaposi's sarcoma) and some showed lesser age advancement (e.g., leukemias and lymphomas), although the groups were too small to demonstrate statistically significant differences (Table 2).

**TABLE 2:** Comparing mean of age delta across patient characteristics using *t*‐test or ANOVA.

Comparison variables	*N*	Age delta (years): mean (SD)	*p*‐value
Race			
Black	16	4.81 (5.19)	0.627
White	80	5.5 (5.15)	
Sex			
Male	88	5.6 (5.19)	0.171
Female	8	3 (3.96)	
Initial ART Era			
II	41	4.8 (5.5)	0.342
I	55	5.82 (4.85)	
Smoking status			
Ever	64	5.9 (4.61)	0.291
Never	31	4.68 (5.96)	
Type of cancer			
Skin	11	3.45 (5.54)	
Anal	7	5.71 (5.53)	
Leukemias/Lymphomas	4	1.5 (6.56)	
GU	4	5.67 (4.16)	
Lung	3	8.67 (2.52)	0.287
Kaposi's sarcoma	3	9.67 (1.15)	
Colorectal	2	0.5 (3.53)	
Esophageal	1	3	
None	61	5.82 (5.09)	
Time from diagnosis to ART			
<2 years	60	4.72 (5.29)	0.1
≥2 years	36	6.5 (4.74)	

Univariate analysis and multivariable linear regression were performed and summarized in Table 3. The univariate analysis showed that the only factor statistically associated with age delta was chronologic age. As the age at the time of HIV diagnosis increased, age delta decreased. Sex, race, tobacco use, ART treatment, and cancer development were not significantly associated with the age delta in this cohort.

**TABLE 3:** Results of univariate and multivariable linear regression predicting age delta.

	Univariate analysis		Multivariable regression	
	Coefficient	*p*‐value	Coefficient	*p*‐value
Chronologic age at diagnosis (per year increase)	−0.47	<0.0001	−0.51	<0.0001
Time from HIV to ARV (≥2 vs. <2 years)	1.78	0.1	1.91	0.011
Race (White vs. Black)	0.69	0.627	−2.74	0.038
Sex (male vs. female)	2.6	0.171	2.97	0.089
Initial ART Era (II vs. I)	−1.01	0.342	−2.64	0.0006
Smoking status (ever vs. never)	1.18	0.291		
Cancer (yes vs. no)	1.19	0.276		
Type of cancer				
Skin versus none	−2.32	0.165		
Anal versus none	−0.06	0.976		
Leukemias/lymphomas versus none	−4.27	0.105		
GU versus none	−1.02	0.696		
Lung versus none	2.89	0.337		
Kaposi's sarcoma versus none	3.89	0.197		
Colorectal versus none	−5.27	0.151		
Esophageal versus none	0	NA		

In multivariable regression analysis predicting age delta with age at diagnosis, race, sex, and ART treatment as model covariates, all were statistically associated with age delta except sex. In the multivariate analysis, Black patients showed a negative correlation with age delta (less age acceleration) compared with White patients. Patients initially treated with ART II therapy showed a negative correlation with age delta compared to those who started with ART I therapy. Patients with delayed initiation of ART treatment (≥2 years) showed a positive correlation with age delta (greater age acceleration) compared to those who received immediate initiation of ART treatment (<2 years). As was seen in the univariate analysis, chronologic age at the time of HIV diagnosis was shown to strongly negatively correlate with age delta.

## 4 DISCUSSION

In this analysis of blood samples from patients with HIV, we identified an ~5‐year DNAm age advancement using the Horvath method. We also observed a reduction in age advancement during extended ART treatment in paired samples collected at least 10 years apart. In this sample set, we did not detect any impact of ART cocktail composition or timing of treatment initiation on the age acceleration pattern.

Our finding of an ~5‐year mean DNAm age advancement is largely consistent with other studies.^20^, ^21^, ^22^ Most similar studies have been carried out in cohorts of PLWH on ART therapy, so it has not been clear whether HIV infection and/or ART are the critical drivers of the advancement. Recent studies have suggested that even early stage HIV infection (prior to significant immunosuppression and initiation of ART) is associated with age acceleration.^34^ In relatively short‐term studies comparing PLWH pre‐ART and post‐ART (up to 24 months) to controls, ART treatments tended to be associated with a reduction in epigenetic age acceleration.^23^ However, the long‐term effects of ART on epigenetic age acceleration have not been assessed. In our study, we obtained paired blood samples from 40 individuals, one at the time of diagnosis (pre‐ART), and another after at least 10 years of ART therapy (post‐ART). Our results showed a mean of ~14 years of age acceleration (age delta) in pre‐ART samples, and a mean of ~6 years of age delta in post‐ART samples (Figure 2). These results indicate that premature aging in PLWH is not accelerated by ART. To the best of our knowledge, this is the first study to examine the long‐term effects of ART on mitigation of epigenetic age acceleration.

The type of ART therapy initiated and timing of treatment relative to HIV diagnosis have been implicated in the premature aging of PLWH.^35^, ^36^ The current ART agents are known for reduced toxicity and side effects, and some HIV‐associated phenotypes are milder. For example, 20% of PLWH developed HIV‐associated dementia (HAD), the most severe form of HIV‐associated neurocognitive disorders (HAND), before 2001. In the ART era II, milder form of HAND are more prevalent.^8^ Here, the multivariable analysis of our cohorts is consistent with these previous observations. Patients that started with both era I and era II ART therapies experienced a reduction in age acceleration (Figure 2), whereas starting with ART era II therapy was negatively correlated with age delta, and delayed initiation of ART was positively correlated with age delta (Table 3). These results provide support for the suggestion that early ART treatment and newer ART regimens may benefit patients.^35^, ^36^

Current DNA methylation‐based methodologies are able to predict age.^16^, ^17^ However, recent studies have shown that these models systematically underestimate age in tissues from older people.^37^ Our study shows that the age delta decreased in patients over 60 years of age (Figure 1B). In a cohort of Black PLWH older than 60 years of age screened in New York City, the average age acceleration was about 2 years,^38^ less than the acceleration reported for the PLWH population at large. Though the New York cohort study did not include younger Black PLWH, it adds to concerns that the current models may not provide an accurate assessment of age acceleration in older individuals. Further study is required to determine whether this is due to the commonality of comorbidities in this population, insensitivity of the analysis, chronologic age‐mediated DNA methylation changes, or other causes.

Recently, an increase in early onset of common cancers was noted in PLWH.^39^ Accelerated epigenetic age is associated with cancer development^29^, ^40^ and cancer also impacts epigenetic age depending on the cancer types.^16^, ^41^, ^42^ Since we had access to a long‐term cohort, we were able to assess the link between cancer, age, and HIV infection to assess whether there was evidence that cancer further accelerated the epigenetic aging in PLWH. Our results showed that cancers did not further increase age delta in PLWH (Figure 3). We observed an increased incidence of cancers in PLWH (Table 1), consistent with data from previous other studies.^3^ From this, although the density of data is slight, we conclude that premature aging caused by HIV infection may be a risk factor for cancer, however, the development of cancer does not further increase epigenetic age acceleration as estimated from peripheral blood.

Smoking has been considered as a critical risk factor for age‐related morbidity and mortality for many decades.^43^, ^44^ Smoking accelerates the epigenetic age of human respiratory organs including airway cells and lung tissue, and smoking cessation reduces the methylation level of airway cells to that of nonsmokers.^45^ However, the effect of smoking on age acceleration based on the methylation level of blood samples is not clear^18^; some studies have employed a separate set of loci to analyze age acceleration derived from blood samples.^46^ Smoking prevalence is higher among PLWH than among the general population and smoking in PLWH further increases the lung cancer risk compared to PLWH nonsmokers.^47^ Our results showed that smoking status was not differentially associated with age delta in our cohort of patients (Tables 2 and 3). This result suggests that smoking mainly affects the epigenetic aging of the respiratory system and the effects on the epigenetic aging of blood do not appear to be significant.

Our study has several limitations, including a cohort of mostly White men, with limited numbers of Black men and women. Therefore, the comparisons of age acceleration across race and sex were limited by the small sample sizes. This study lacks sample size estimation, though the number of the subjects is within the range of other published studies.^20^, ^21^ In addition, the retrospective study design limits our ability to differentiate the effects of each ART era on age acceleration. Future studies will need to utilize samples collected at variable intervals in the natural history of HIV infection to address the long‐ and short‐term effects of specific therapies on age advancement. In addition, it would be valuable to add this assessment to determine if links exist between chronic inflammation, various immune subtypes, and tissue and blood cell methylation markers in future studies.

In conclusion, our study demonstrates that the age acceleration using mDNA in PLWH is mitigated by prolonged ART therapy and is not further accelerated by a diagnosis of cancer.

## AUTHOR CONTRIBUTIONS

**Yulan Qing:** Conceptualization (equal); data curation (lead); formal analysis (lead); investigation (lead); methodology (supporting); project administration (equal); writing – original draft (lead); writing – review and editing (lead). **Ricky Chan:** Formal analysis (equal); investigation (equal); methodology (equal); software (equal); writing – review and editing (equal). **Pingfu Fu:** Formal analysis (equal); investigation (equal); methodology (equal); software (equal); writing – original draft (equal); writing – review and editing (equal). **Jennifer Cullen:** Formal analysis (equal); investigation (equal); methodology (equal); software (equal); writing – original draft (equal); writing – review and editing (equal). **Alexander Miron:** Formal analysis (equal); investigation (equal); methodology (equal); writing – review and editing (equal). **Jeffrey M. Jacobson:** Investigation (equal); resources (equal); writing – review and editing (equal). **John Pink:** Conceptualization (equal); formal analysis (equal); investigation (equal); methodology (equal); writing – original draft (equal); writing – review and editing (equal). **Stanton L. Gerson:** Conceptualization (equal); data curation (equal); funding acquisition (equal); investigation (equal); resources (equal); supervision (equal); writing – original draft (equal); writing – review and editing (equal).

## FUNDING INFORMATION

This work was supported by National Institutes of Health USA (P30AI036219, P30CA043703, and 5U54CA254566‐02).

## CONFLICT OF INTEREST STATEMENT

The authors have no conflicts of interest to report.

## Supporting information


Table S1

## Data Availability

Data in this study will be available upon request.
