# Supplementary material for: Impact of age, antiretroviral therapy, and cancer on epigenetic aging in people living with HIV
Source: Cancer Med. 2023 Mar 23;12(9):11010–9. doi: 10.1002/cam4.5809 (PMC10225243; doi:10.1002/cam4.5809)
Supplement: Supplementary file 1 — Table S1 [file CAM4-12-11010-s001.docx]

**Table S1. Interval between cancer development and HIV diagnosis**

| **Comparison Variable** | **N** | **Time (years):** Mean (STD) |
| --- | --- | --- |
| **Time of cancer diagnosis** |  |  |
| Before HIV diagnosis | 2 | -4 |
| At the time of HIV diagnosis | 5 | 0 |
| After HIV diagnosis | 27 | 14.41(8.03) |
| unknown | 1 |  |
|  |  |  |
| **Cancer subtype** |  |  |
| Skin | 11 | 13.44(5.80) |
| Basal cell | 7 | 12.42(5.74) |
| Squamous cell | 2 | 13.23 |
| Melanoma in situ | 1 | 22.07 |
| Anal | 7 | 10.40(13.01) |
| Squamous cell | 6 | 10.54(14.24) |
| In situ | 1 | 9.54 |
| Leukemias/Lymphomas | 4 | 10.15(11.12) |
| Acute myelogenous leukemia | 1 | 24.83 |
| Burkitt's lymphoma | 2 | 7.90 |
| Diffuse large cell B lymphoma | 1 | 0 |
| GU | 4 | 9.67(12.20) |
| Prostate | 2 | 16.98 |
| Testicle | 1 | -6.42 |
| Bladder in situ | 1 | 11.12 |
| Lung | 3 | 18.30(9.55) |
| Kaposi’s Sarcoma | 3 | 1.54(2.79) |
| Colorectal | 2 | 10.73 |
| Esophageal | 1 | unknown |
